# Changes in Cortical Thickness in 6-Year-Old Children Open Their Mind to a Global Vision of the World

**DOI:** 10.1155/2014/362349

**Published:** 2014-07-09

**Authors:** Nicolas Poirel, Elise Leroux, Arlette Pineau, Olivier Houdé, Grégory Simon

**Affiliations:** ^1^LaPsyDÉ, UMR 8240, CNRS, Université Paris Descartes and Université de Caen, Sorbonne, 46 rue Saint-Jacques, 75005 Paris, France; ^2^Institut Universitaire de France, 75005 Paris, France; ^3^ISTS, UMR 6301, CNRS, CEA, 14000 Caen, France; ^4^CHU de Caen, Service de Psychiatrie, Centre Esquirol, 14074 Caen, France

## Abstract

Even if objectively presented with similar visual stimuli, children younger than 6 years of age exhibit a strong attraction to local visual information (e.g., the trees), whereas children older than 6 years of age, similar to adults, exhibit a visual bias toward global information (e.g., the forest). Here, we studied the cortical thickness changes that underlie this bias shift from local to global visual information. Two groups, matched for age, gender, and handedness, were formed from a total of 30 children who were 6 years old, and both groups performed a traditional global/local visual task. The first group presented a local visual bias, and the other group presented a global visual bias. The results indicated that, compared with the local visual bias group, children with a global visual bias exhibited (1) decreased cortical thickness in the bilateral occipital regions and (2) increased cortical thickness in the left frontoparietal regions. These findings constitute the first structural study that supports the view that both synaptic pruning (i.e., decreased cortical thickness) and expansion mechanisms (i.e., increased cortical thickness) cooccur to allow healthy children to develop a global perception of the visual world.

## 1. Introduction

**Figure 1:**
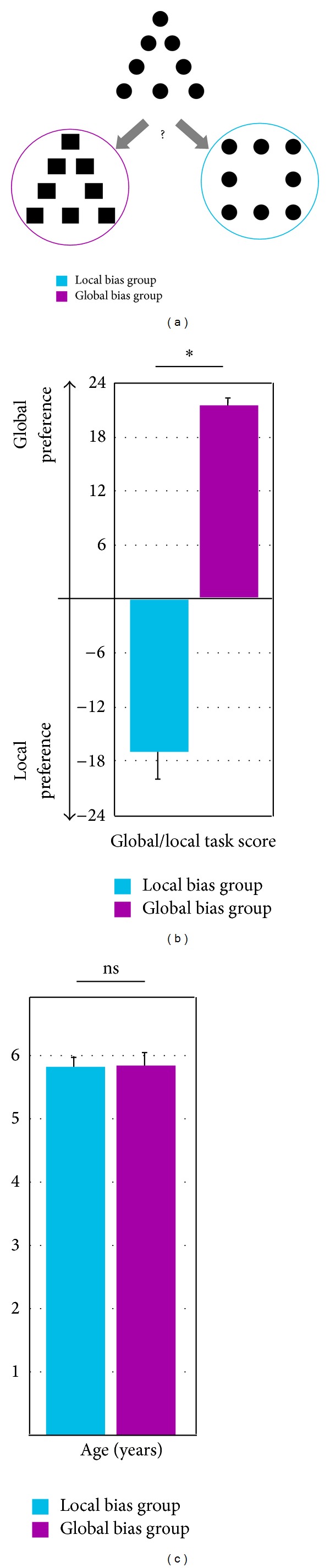
Representative examples of global/local triad stimuli (a), mean scores for the global/local task (b), and mean ages (c) of the local bias group (blue) and the global bias group (pink). *P < 0.05, ns: nonsignificant.

Adults and children do not equally perceive the forest (i.e., global visual information) and the trees (i.e., local visual information). Even if objectively presented with similar visual stimuli, children younger than 6 years of age exhibit a strong attraction to local information, whereas children older than 6 years of age exhibit, similar to adults [1–3], a visual attention bias toward global information [4–6]. Because the global level (e.g., the whole, the forest) can be predicted from the identity of the local level (e.g., the features, the trees) and viceversa in a real-word situation, experimental materials that included a global level that could be apprehended independently of the local level (and vice versa) were developed by Navon [2, 7]. These compound stimuli consisted of large global forms composed of small local elements (e.g., a global triangle composed of local circles; see Figure 1) that presented an elegant method for performing global/local studies. First, the set of possible global features is identical to the set of possible local features (i.e., both could represent any possible geometric form). Second, the independence of the global and local features is left intact, such that the geometric form presented at the global level cannot be predicted from the identity of the geometric form presented at the local level and vice versa. When children were presented with compound stimuli and asked to draw them from memory, Dukette and Stiles [8] showed that (1) global visual processing was not as fully developed in younger children and (2) compared with adults the younger children were biased toward the local level. An age-related change in global/local processing was proposed to be mediated by evolution of the visuospatial strategy employed by children [4, 9, 10]. In particular, after the age of 6 years, children exhibit more exploratory eye movements than younger children, suggesting a shift from a local sampling strategy of visual information to a more exhaustive exploration of global visual stimuli [10, 11].

The visuospatial proficiency for global visual information was also suggested to cooccur with the development of a hemispheric specialization for global/local processes in children [12]. Indeed, seminal neuropsychological studies in children [13] and adults [14] have indicated that the left and right hemispheres are biased toward local and global visual processes, respectively. Consequently, unilateral lesions in the left or right temporoparietal cortex impair the underlying attentional and perceptual mechanisms associated with local and global processes, respectively [15]. These findings were confirmed using functional brain imaging in healthy adults [16–18] and 14-year-old children [19]. The results showed hemispheric specialization in the visual areas in the right middle occipital cortex, which was more active during the global tasks than the local tasks, and in the left inferior occipital cortex, which was more active during the local compared with global tasks [17]. Hemispheric asymmetries in the temporoparietal regions during global/local processes are also supported by neuroimaging studies [18, 20], suggesting that the parietal regions may be critical for shifting attention from one level of process to another [21]. In children, the use of anatomical voxel-based morphometry methods revealed a cooccurrence of gray matter modulation in these aforementioned regions and the emergence of selective specialization for global visual processing [5]. In particular, compared with 6-year-old children with a local visual bias, 6-year-old children with a global visual bias exhibited gray matter loss in the right inferior occipital cortex (extending to the middle occipital gyrus), the right parietal precuneus, and the right precentral gyrus. This loss in gray matter density is traditionally attributed to a reduction in synaptic density, a phenomenon called “synaptic pruning,” which is a fundamental neural plasticity mechanism that underlies selective behavioral specialization [22]. Consequently, the gray matter loss in the right hemisphere in 6-year-old children suggests the fine tuning of a brain network for the processing of global visual information. Taken together, these results underscore the fact that the emergence of an occipitoparietal brain network at the age of 6 years allows access to the essential capacity to consider all global information present in a visual environment. However, no studies performed to date have uncovered changes in cortical thickness that enable this shift from local to global visual processing at approximately 6 years of age. Although it has been recently shown that cognitive abilities are strongly linked to the dynamics of cortical thickness [23], no studies have, to the best of our knowledge, investigated cortical thickness modifications during the well-known developmental period in which the mode of visual attention changes from a local to a global bias. The current study used a sulcogyral parcellation method that provides a measure of the thickness of each surface according to the Destrieux et al. Atlas [24]. Outside the scanner, the children performed a classical global/local task that allows the determination of their visual bias (i.e., global or local; see [5, 6, 25, 26]). Anatomical magnetic resonance imaging (MRI) images of each child were also acquired to determine whether the shift from a local to a global visual processing bias corresponded to changes in gray matter thickness. Because cortical thickness exhibits a general linear decrease with development [27], we hypothesized that the cortical thickness in the global bias group would be decreased compared with that in the local bias group. In particular, we expected that compared with the children with a local visual bias in the experimental task (i.e., local bias group), the children with a global visual bias (i.e., global bias group) would exhibit modulation primarily in the right occipitoparietal network, which is strongly implicated in global processing in adults [17, 18] and children [5]. We did not expect differences between the two groups of children in the left hemisphere (involved in local visual processing, e.g., [16]), as the capacity necessary to process local information appears to be efficient as early as 3 years of age [28]. To test these hypotheses, we compared anatomical MR images between 6-year-old children who presented either a local or a global visual processing bias. In agreement with the principle of selective specialization, we hypothesized that the reduction of cortical thickness in the right hemisphere in children in the global bias group would be associated with the emergence of an adult-like global attentional mode of visual processing.

## 2. Methods

### 2.1. Participants

Thirty children from Caen (Calvados, France) participated in this study (mean ± standard deviation (M ± SD), 5 years 11 months ±  7.4 months; 20 girls; 25 right-handed). The children had no history of neurological disease and no cerebral abnormalities, as assessed by T_1_-weighted MRI. The local ethics committee approved the study. Written consent was obtained from the parents and the children themselves after a detailed discussion and explanations (individual consent of the children was indicated by a smiley face associated with a specific color).

### 2.2. MRI Acquisition and Analysis

Anatomical images were acquired for each child on a 3-Tesla MRI scanner (Intera Achieva, Philips Medical System, The Netherlands) using 3D T_1_-weighted spoiled gradient images (field of view [FOV]: 256 mm; slice thickness: 1.33 mm; number of slices: 128; matrix size: 192 × 192 voxels; duration: 5 min 7 s). Brain images were acquired, while the children passively watched a cartoon on an MRI-compatible screen. The sedative impact of audio/visual systems on children in an MRI scanner has been demonstrated previously; specifically, the systems reduce motion, provide a positive experience, and decrease wait times [29].

Cortical thickness estimation from 74 brain regions per hemisphere was performed for each participant using the Freesurfer 5.1 analysis suite with Destrieux et al.'s Atlas [24] (documented and freely available for download online, http://surfer.nmr.mgh.harvard.edu/). The technical details of the procedures were described previously [30–32]. For processing, we used optimized intensity nonuniformity correction for 3 Tesla MRI scanners [33] and a process that included visual inspections and the manual correction of topological defects.

### 2.3. Local/Global Task

After the laboratory MRI session, all children were presented with the global/local task at school. A total of 24 compound stimulus trials were presented to measure the global/local perceptual bias. Children judged which of the two comparison figures was most similar to a reference (Figure 1(a)). The judgment could be based on either the local or global aspect of the reference. Children were instructed to give their first, most immediate similarity judgment for each trial. A measure of global/local precedence was then calculated for each participant by subtracting the number of local choices from the number of global choices. The scores ranged between −24 and 24. A positive score indicated a greater predilection toward the global information, whereas a negative score indicated a greater predilection toward the local information. Two groups of children were formed according to their local/global score. Children with negative scores were included in the local bias group, and children with positive scores were included in the global bias group.

## 3. Results

### 3.1. Behavioral Results

Ten children presented a local bias (8 girls, 9 right-handed, score on the global/local task: −17.6 ± 2.8), and 20 children presented a global bias (12 girls, 16 right-handed, score on the global/local task: 21.8 ± 0.8). Ten children were thus selected from the global bias group and were matched for gender (8 girls) and handedness (9 right-handed) to the local bias group (score on the global/local task: 21.6 ± 1; see Table 1). The scores in the local bias group and the global bias group differed significantly (*P* < 0.0001, Figure 1(b)). Importantly, the age between the two groups did not differ (*P* = 0.85; see Figure 1(c)). Note that all analyses presented hereafter were also performed with all participants (i.e., local bias children, *n* = 10, versus global bias children, *n* = 20). The results were similar to those obtained when comparing the two matched groups of children.

### 3.2. Cortical Thickness Analyses

Mean cortical thickness values of each area extracted from Freesurfer software were compared between the two groups of children (i.e., local group and global group) using *t*-tests with JMP software. Note that as in previous neuroimaging studies that included a limited number of children [34–36] or adults [37], cortical thickness variations were reported when mean values were significantly different at *P* < 0.05 uncorrected.

**Figure 2:**
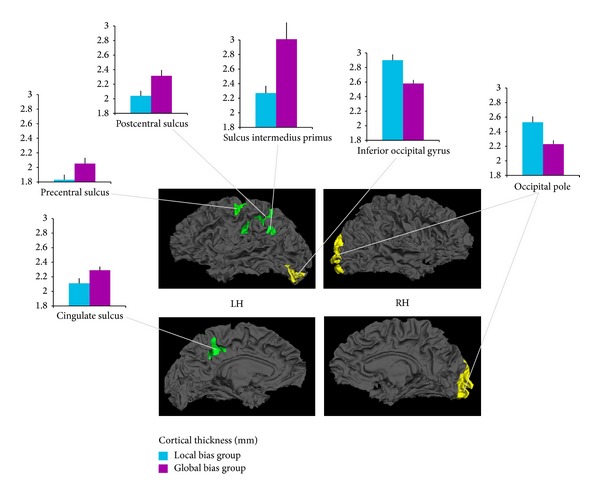
3D rendering of local brain regions showing significant decreases (yellow) and increases (green) in cortical thickness (in mm) between children in the local bias group and the global bias group. LH: left hemisphere; RH: right hemisphere.

The analyses of cortical thickness revealed cortical thickness decreases in the left inferior occipital gyrus (*t* = 3.32, *P* = 0.004) and the right occipital pole (*t* = 3.17, *P* = 0.005) in the global bias group compared with the local bias group. Moreover, an increased cortical thickness was observed in the left regions, including the postcentral sulcus (*t* = 2.58, *P* = 0.02), the superior region of the precentral sulcus (*t* = 2.21, *P* = 0.04), the marginal branch of the cingulate sulcus (*t* = 2.17, *P* = 0.04), and the sulcus intermedius primus of Jensen (in the inferior parietal lobe) (*t* = 2.99, *P* = 0.008, Figure 2), in the global bias group compared with the local bias group.

## 4. Discussion

The present study is the first to document variations in cortical thickness during the developmental window corresponding to a shift from a local visual processing bias to an adult-like global visual processing bias. A clear difference in visual performance was observed in 6-year-old children, with two subgroups of children presenting either a local or a global visual bias. This difference in behavioral performance observed at 6 years of age was in agreement with the traditional visuospatial shift observed at this developmental window [4, 5]. Using a well-validated analytical approach to measuring cortical thickness in the brain [30, 31], we demonstrated that compared with the local visual bias group, children with a global visual bias had (1) decreased cortical thickness in the bilateral occipital regions and (2) increased cortical thickness in the left frontoparietal and cingulate regions. These changes, demonstrated for the first time in healthy children, are consistent with those observed using functional brain-imaging techniques in adults, particularly for brain regions such as the occipital cortex [16, 17] and the attentional parietal regions [18, 20]. In agreement with the hypothesis that synaptic pruning occurs with the specialization for global processing in children [5], we observed decreased cortical thickness in the occipital regions. These primary visual areas are known to be strongly involved in global/local processing in adults, both in the left and right hemispheres [16, 17, 38]. Interestingly, and in line with our previous anatomical brain results in children [5], the variation in gray matter thickness was more important in the right hemisphere (occipital pole, 23.43 cm^2^; see [24]) than in the left hemisphere (inferior occipital gyrus, 13.22 cm^2^; see [24]). This difference could reflect the more selective specialization of the early stages of visual processing in the right hemisphere that has to be initiated before the shift to a global visual bias in children can occur. In particular, because the right occipital regions are known to be devoted to global information processing in adults [17], it is conceivable that the emergence of a global visual bias in children leads to a stronger synaptic pruning plasticity phenomenon in the right occipital regions than in the left occipital regions. Consequently, the selective specialization of the left early visual area, which is known to be more important during local visual processing [16], was less pronounced than that in the right early visual area. This assumption agrees with the view that children 6 years of age already have strong abilities to process local visual information [39] and that early local visual processes appear to be present as early as 3 years of age [28] and are stable by approximately 4 years of age [8, 40]. Taken together, the decrease in cortical thickness in the bilateral occipital regions suggests a fine-tuning of the primary visual cortex in children (more pronounced in the right hemisphere) due to the emergence of a global visual bias at the age of 6 years.

However, increased cortical thickness in the left frontoparietal and cingulate regions was also observed in children with a global visual bias (compared with the immature local visual bias). These unexpected but very interesting findings suggest that the synaptic pruning hypothesis is not compatible with the cortical thickness variations observed in the prefrontal, parietal, and cingular regions in 6-year-old children. Alternatively, this increase in cortical thickness could be interpreted as a possible preliminary expansion mechanism of a brain network that initially allows children to shift from a local to a global visual bias. The primary sensory areas, such as the primary visual cortex, are the first to show a maturation of thickness (characterized by a decrease in cortical thickness), followed by the parietal and prefrontal regions [41, 42], demonstrating a posteroanterior gradient in brain development. We thus suggest that the primary visual areas could be the first to present a decrease in cortical thickness (that cooccurs with the emergence of the global visual bias in children), whereas the parietal, prefrontal, and cingulate regions have to be strongly solicited during this transitional period to inhibit the automatic visual processing bias toward local information (i.e., the previous visual bias in children). This assumption is compatible with recent findings in adults that showed the involvement of attentional and executive inhibition processes associated with global level information (i.e., the dominant mode of visual information in adults) during local processing (i.e., the nondominant visual information in adults; see [43]). The posterior parietal cortex (along the postcentral sulcus, as found in the present work) was also shown to be involved in attentional control during global/local processing [44]. In particular, Draganski et al. [45] showed that the parietal cortex represented a core region that allowed efficient biasing of the attentional focus away from the salient characteristics of a visual stimulus. Consequently, during the transitional period at 6 years of age, children may have to revert to the salient local predominant information (i.e., their dominant “visual default mode strategy”) to correctly consider the new visual bias toward global information that arises at this age (due to the decreased occipital cortical thickness). Thus, the new global visual bias in children could lead to an increase in cortical thickness in the left lateralized brain network (see, for instance, [45] for a discussion on the expansion mechanisms), which would enable disengagement from the automatic attentional focus on local information. Importantly, the increase in cortical thickness found in the present study was observed only in the left hemisphere regions, which are known to support local processing [20] and dominate the initial attentional visual mode in children. This network included the parietal (postcentral gyrus and sulcus intermedius primus), frontal (precentral gyrus), and cingulate regions, which were previously shown to be involved in visual attentional control processes in adults [46–48]. The aim of the present study was to elucidate the variation in cortical thickness during the transitional period that corresponds to the shift from a local to a global visual bias; however, further studies are needed to determine whether the parietofrontal network (i.e., the thickness of which increased in the present study) also presents a decrease in cortical thickness (i.e., synaptic pruning) as the global visual bias stabilizes with age (given that it has been suggested that the global visual bias continues to be refined until 9 years of age [4]). A limitation of the present study is the small sample size and the use of uncorrected thresholds. Nevertheless, we note that changes in gray matter thickness corresponding to the shift from a local to a global visual processing bias in children observed in the present study are localized in cortical areas activated in global and local processing in adults [16–18, 20, 38, 44].

In conclusion, the present findings provide the first evidence of a direct relationship between the emergence of a global visual bias and the variation in cortical thickness in children. The data reported here indicate that, compared with children with a local visual bias, children with a global visual bias are characterized by both decreases and increases in cortical thickness in the occipitoparietofrontal brain network. These findings constitute the first structural study that supports the view that both synaptic pruning (i.e., decreased cortical thickness) and expansion mechanisms (i.e., increased cortical thickness) cooccur to allow healthy children to develop a global perception of the visual world.

## Figures and Tables

**Table 1:** Characteristics of the local bias group and the global bias group of children.

	Local bias group (*n* = 10)		Global bias group (*n* = 10)		
Gender (F/M)	8/2		8/2		
Handedness	9 right-handed		9 right-handed		

	Mean	(SD)	Mean	(SD)	P*

Age (mean: years; months, SD: months)	5; 10	(6)	6; 1	(8)	0.85
Local/global task score (min/max: −24/+24)	−17.6	(2.8)	21.6	(1)	<0.0001

*P values: *t*-tests.

SD: standard deviation; F/M: female/male.
